# Phenotypic and Nutritional Diversity Reveal Elite Accessions of *Berberis darwinii* Supporting Berry Breeding and Functional Food Applications

**DOI:** 10.3390/plants15071061

**Published:** 2026-03-30

**Authors:** Manuel Chacón-Fuentes, César Burgos-Díaz, Karla Garrido-Miranda, Fernando Westermeyer, Alan Mercado

**Affiliations:** 1 Agriaquaculture Nutritional Genomic Center, CGNA, Temuco 4781158, Chile; cesar.burgos@cgna.cl (C.B.-D.); karla.garrido@ufrontera.cl (K.G.-M.); fernando.westermeyer@cgna.cl (F.W.); alan.mercado@cgna.cl (A.M.); 2 Universidad de La Frontera, Scientific and Technological Bioresource Nucleus (BIOREN-UFRO),Temuco 4780000, Chile

**Keywords:** polyphenols, anthocyanins, antioxidant capacity, fruit biochemical profiling, common-garden experiment, multivariate analysis, low-input cultivation

## Abstract

*Berberis darwinii* is a native Chilean berry distributed across contrasting agro-ecological zones, highlighting its broad ecological amplitude and agronomic relevance. The objective of this study was to identify productive, functional, and balanced elite accessions of *B. darwinii* by integrating phenotypic, fruit quality, nutritional, and antioxidant traits under contrasting water availability. Ninety-six accessions were evaluated in a common-garden experiment over two consecutive growing seasons (irrigated and rainfed) for morphological, productive, and fruit quality traits. Substantial variation was observed in plant height, shoot number, leaf area, and spine density. Across seasons, some accessions combined high yields (up to 8.5 t ha^−1^), fruit diameters exceeding 8 mm, and elevated soluble solids (up to 33 °Brix). Because water regime, season, and plant age were not experimentally separated, these contrasts indicate adaptive performance under contrasting water availability rather than direct irrigation effects. Functional analyses revealed high biochemical diversity, with total polyphenols reaching 18,168.7 mg GAE 100 g^−1^ dry weight, anthocyanins up to 5747.7 mg cyanidin-3-glucoside 100 g^−1^ dry weight, and ORAC values up to 35.4 mmol Trolox 100 g^−1^ fresh weight. Multivariate analyses supported the selection of elite candidates for low-input domestication and functional ingredient development. This integrated common-garden framework links intra-specific phenotypic variation with phenolic/antioxidant diversity, supporting trait-based selection and interpretation of stress-associated secondary metabolism.

## 1. Introduction

Chilean Patagonia hosts a wide diversity of habitats and vascular plants, including numerous wild edible berries [1,2]. International interest in South American native berries has increased, particularly in the context of functional foods and crop adaptation under changing climatic conditions [1,2,3]. Fruits such as Michay (*Berberis darwinii*), Calafate (*B. microphylla*), Maqui (*Aristotelia chilensis*), and Murta (*Ugni molinae*) have historically been consumed as foods [4,5]. Within this group, the Berberidaceae family is notable for its ecological adaptability, represented in South America exclusively by Berberis [5,6,7]. *Berberis darwinii*, known as “Michay” [8,9] or “Quelung” [10], is native to temperate forests of southern South America, occurring from Ñuble to Aysén in Chile and in mountainous areas of Argentine Patagonia [11]. This species reaches 1–3 m in height and produces dark-pigmented berries with documented antioxidant and nutraceutical potential [9]. Despite growing interest, the biochemical and nutritional characterization of Chilean native berries remains limited [12,13]. In particular, information on the extent of phenotypic, productive, and biochemical variability of *B. darwinii* under cultivation is scarce, limiting its evaluation as a candidate crop. Research on native berries is increasingly focused on their natural strategies to cope with environmental variability [14], especially under scenarios of increasing drought and low-input agriculture. As global demand for sustainable production systems rises, identifying antioxidant-rich berry crops suitable for low-input environments has become essential for food security [15,16,17,18]. Several Chilean native berries have been proposed as functional foods [18,19,20,21,22], linked to their ability to neutralize free radicals and counteract oxidative stress [22]. Recent reviews highlight the biotechnological potential of Berberis fruits, particularly *B. darwinii*, due to their high phenolic content [7]. Michay concentrates polyphenols, mainly anthocyanins in the fruits, as well as other phytochemicals in stems, leaves, and roots [23,24], supporting strong antioxidant capacity [25]. Antioxidant levels in Michay have been reported to be comparable to or higher than those of other native berries, including Calafate [18]. Phenolic-enriched extracts of wild Michay have demonstrated ORAC activity of 2000–3000 μmol TE g^−1^, exceeding Calafate (840–2900 μmol TE g^−1^) and strawberry (*Fragaria chiloensis*) (770–810 μmol TE g^−1^) [12]. However, these reports are based exclusively on wild populations. No studies have systematically evaluated antioxidant content, phenotypic traits, and nutritional properties of cultivated Michay under controlled field conditions. Preliminary observations describe *B. darwinii* as tolerant to drought, frost, shade, and diverse soil types [26,27,28]. While these observations suggest broad ecological adaptability, experimental validation under cultivation remains limited, particularly regarding fruit production and quality. Water scarcity is increasingly threatening fruit production worldwide [29]. Conventional antioxidant-rich berries such as blueberry (*Vaccinium corymbosum*) show high drought sensitivity due to shallow root systems [30,31] and strong irrigation dependence [32]. Native Chilean berries also display drought sensitivity: full irrigation suppression reduced fruit number by 30% and yield by 20% in *U. molinae* [33]; *B. microphylla* requires 450–550 mm of seasonal irrigation [34]; and *A. chilensis* suffers high plant mortality under reduced irrigation [35]. Although moderate water stress may increase antioxidant accumulation [36,37], it often compromises plant growth and productivity. To date, few studies have experimentally evaluated Chilean native berry species under common-garden field conditions across contrasting seasonal water availability, particularly in terms of jointly assessing productivity and fruit quality. Despite increasing recognition of the functional and agronomic potential of *B. darwinii*, critical knowledge gaps remain. Existing studies on native Chilean berries have typically focused on isolated dimensions, such as antioxidant profiling of wild populations or descriptive yield assessments, without integrating phenotypic, productive, nutritional, and biochemical traits within a common-garden experimental framework. In particular, no study has systematically evaluated intra-specific diversity under cultivation while simultaneously examining productive performance and antioxidant-related compounds under contrasting seasonal water availability. Moreover, the identification of elite accessions suitable for breeding programs or low-input cultivation strategies has not been supported by multivariate trait integration approaches capable of capturing trade-offs and complementary trait syndromes relevant for functional food applications. By integrating common-garden evaluation, biochemical profiling, and multivariate analysis under contrasting seasonal water availability, this study moves beyond single-trait approaches and introduces a structured framework for elite accession identification and sustainable berry breeding. Importantly, the contribution is not limited to identifying high-performing accessions; rather, we use a common-garden multi-trait framework to characterize how productive, quality, and phenolic/antioxidant traits co-vary within a species. This trait-coordination perspective provides plant-science insight into intra-specific metabolic diversity and stress-associated phenolic variation relevant to trait-based selection. Based on these gaps, this study aimed to identify elite accessions of *B. darwinii* using an integrative multi-trait framework combining morphological, productive, nutritional, and antioxidant variables evaluated over two consecutive growing seasons with contrasting water availability. Rather than maximizing single traits, this approach provides experimental evidence of phenotypic plasticity, adaptive performance, and trait integration in Michay, establishing a foundation for its development as a promising crop for berry breeding and low-input production systems. This study therefore not only supports selection of elite accessions but also contributes to understanding intra-specific phenolic variability and antioxidant responses under contrasting environmental conditions in perennial native shrubs.

## 2. Results

### 2.1. Morphological Traits Variation 

**Figure 1:**
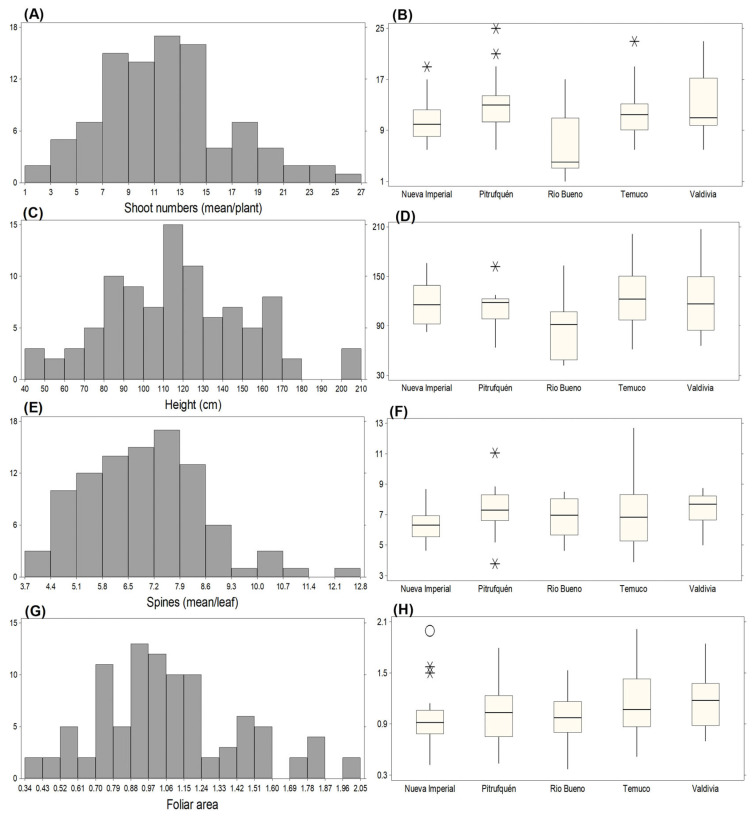
Distribution and variation of morphological traits across different locations. (**A**) Frequency distribution of shoot numbers. (**B**) Shoot numbers across locations. (**C**) Frequency distribution of plant height. (**D**) Plant height across locations. (**E**) Frequency distribution of spine numbers. (**F**) Spine numbers across locations. (**G**) Frequency distribution of foliar area. (**H**) Foliar area across locations. Extreme values are indicated as “*” (possible outliers) and “O” (probable outliers), as defined in the statistical analysis section.

The evaluation of 96 *B. darwinii* accessions revealed substantial phenotypic diversity in vegetative morphology (Table 1). Shoot number showed the greatest dispersion (mean = 11.32 shoots plant^−1^, CV = 42.60%; range = 1–25), evidencing strong heterogeneity in vegetative vigor. Plant height displayed moderate variation (mean = 116.36 cm, CV = 30.19%; range = 42–207 cm), whereas foliar area also varied widely (mean = 1.07 cm^2^, CV = 34.06%; range = 0.37–2.01 cm^2^). In contrast, spine number per leaf exhibited comparatively lower variability (mean = 6.96 spines, CV = 23.11%; range = 3.80–12.70), suggesting greater trait stability relative to other vegetative attributes. The distributions illustrated in Figure 1 corroborate these trends.

Most accessions produced 9–15 shoots (Figure 1A), with unusually high shoot numbers observed in Nueva Imperial (C4P17, 19 shoots), Pitrufquén (C2P8, 25 shoots; C4P12, 21 shoots), and Temuco (C3P19, 23 shoots) (Figure 1B). In Valdivia, C1P12 produced 23 shoots but was not classified as an outlier. Accessions C2P8, C3P19, and C1P12 consistently ranked among those with the highest shoot production, whereas C4P5 produced only one shoot, indicating extremely low vegetative vigor (Figure 1B). Plant height distributions showed that most accessions ranged between 110 and 120 cm (Figure 1C,D), consistent with plant stature compatible with manual and semi-mechanized harvesting systems. The tallest accessions were C1P14 (207 cm) and C2P15 (204 cm) from Valdivia, C1P23 (201 cm) from Temuco, and C1P2 (162 cm) from Pitrufquén; these were the only individuals exceeding 2 m. The shortest accession was C1P8 (42 cm) (Figure 1D). Spininess showed lower phenotypic variation than other morphological traits. Most accessions produced 7.2–7.9 spines per leaf (Figure 1E), whereas lower values (3.7–4.4 spines) occurred in a subset of accessions, which may facilitate harvesting. C2P9 (Nueva Imperial) exhibited the highest outlier value (11.05; Figure 1F), while the maximum observed value was 12.7 in C3P19; the lowest values were recorded for C2P14 (3.8) and C3P20 (3.9). Foliar area followed a broad distribution. Most accessions exhibited leaf areas between 0.7 and 1.24 cm^2^ (Figure 1G), while values approaching 2.0 cm^2^ may be advantageous due to their potential contribution to photosynthetic capacity. Outliers were detected in Nueva Imperial (C3P1, 1.99; C2P16, 1.57; C2P12, 1.5), whereas high but non-outlier values occurred in Pitrufquén (C1P1, 1.79), Río Bueno (C2P3, 1.53), Temuco (C1P25, 2.01), and Valdivia (C3P9, 1.84) (Figure 1G). The largest foliar area was observed in C1P25 (2.03 cm^2^), and the smallest in C4P6 (0.36 cm^2^) (Figure 1H). Reported leaf areas of 0.7–4.2 cm^2^ overlap with the 0.3–2.0 cm^2^ range observed in this study, and the previously reported maximum plant height (1.5 m) was exceeded by several accessions surpassing 2.0 m. Collectively, these results demonstrate substantial morphological diversity and plasticity in *B. darwinii* under cultivation, providing a structural basis for subsequent analyses integrating productive and functional traits.

### 2.2. Productive Traits Variation

Given the high variability detected in morphological traits, productive performance was evaluated by analyzing coefficients of variation across the two assessment seasons (Table 2). Mean yield increased from 1021.40 kg ha^−1^ in 2021/2022 to 1285.10 kg ha^−1^ in 2022/2023 season, while variability remained substantial but decreased (CV = 142.89% to 112.97%). Maximum yield reached 8526.60 kg ha^−1^ in 2021/2022 season and 8334.30 kg ha^−1^ in 2022/2023 season, whereas minimum yield was 0.00 kg ha^−1^ in both seasons, confirming the presence of non-productive accessions under common-garden conditions. Fruit diameter increased from 6.15 mm in 2021/2022 to 6.81 mm in 2022/2023, with consistent moderate variability (CV = 13.11% and 13.40%). Minimum diameter increased from 4.07 mm to 4.97 mm and maximum values rose slightly from 8.00 mm to 8.29 mm, indicating an overall shift toward larger fruits in the second season. Soluble solids (°Brix) showed the most pronounced increase, rising from 16.31 °Brix in 2021/2022 to 21.55 °Brix in 2022/2023, accompanied by a marked reduction in variability (CV = 38.22% to 25.88%).

Minimum °Brix increased from 4.23 to 8.00 and maximum values increased from 29.00 °Brix to 33.37 °Brix, reflecting higher soluble solids across accessions in 2022/2023. Fruit number per plant also increased substantially, from 1389.80 fruits in 2021/2022 to 1907.20 fruits in 2022/2023, while variability decreased (CV = 122.45% to 95.84%). Maximum fruit number remained similar across seasons (7017 vs. 7120), whereas the minimum increased from 0.00 to 3.00 fruits plant^−1^, suggesting fewer completely non-fruiting individuals in 2022/2023. Overall, coordinated increases in yield, fruit diameter, soluble solids, and fruit number in 2022/2023 indicate enhanced productive performance and fruit quality relative to 2021/2022. To avoid causal misinterpretation, it should be emphasized that season-to-season differences represent contrasting seasonal water availability and plant developmental stage, rather than isolated, independently replicated irrigation treatment effects. Nevertheless, the concurrent reduction in coefficients of variation across most productive traits suggests a more consistent performance among a subset of accessions, supporting subsequent identification of elite productive genotypes.

#### 2.2.1. Evaluation of Productive Traits Under Irrigated Conditions

**Figure 2:**
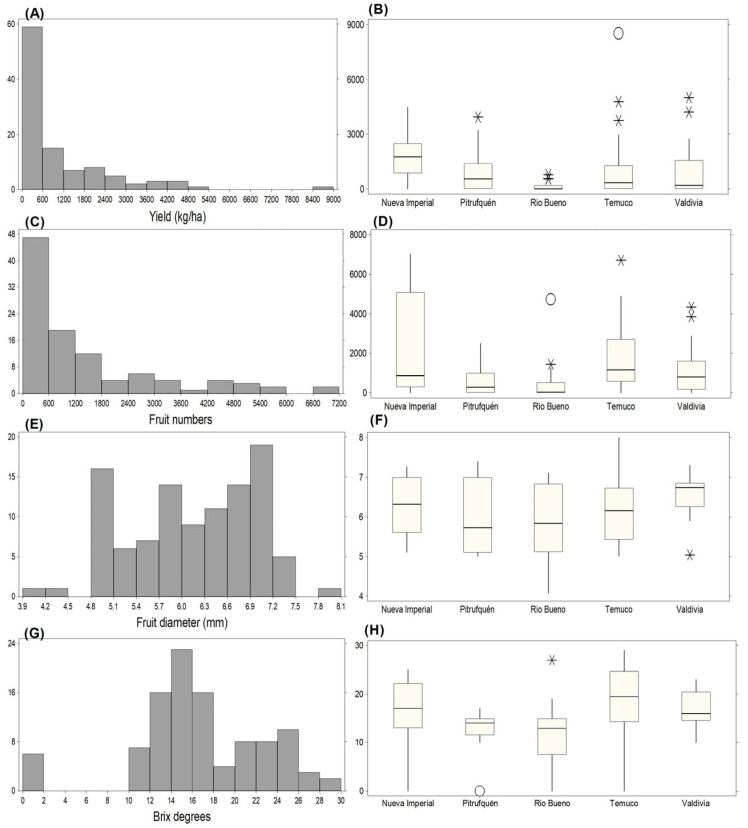
Distribution and variation of yield and fruit traits across different locations on 2021/2022 season. (**A**) Frequency distribution of yield. (**B**) Yield across locations. (**C**) Frequency distribution of fruit numbers. (**D**) Fruit numbers across locations. (**E**) Frequency distribution of fruit diameter. (**F**) Fruit diameter across locations. (**G**) Frequency distribution of Brix degrees. (**H**) Brix degrees across locations. Extreme values are indicated as “*” (possible outliers) and “O” (probable outliers), as defined in the statistical analysis section.

This section describes the distribution and variability of productive traits during the irrigated 2021/2022 season. All productive variables showed substantial dispersion, indicating marked heterogeneity among accessions under common-garden conditions (Figure 2). Yield per hectare exhibited a strongly skewed distribution (Figure 2A,B), with most accessions producing <1000 kg ha^−1^ and a limited number of genotypes deviating markedly from the population. The maximum yield in Nueva Imperial was 3932.08 kg ha^−1^ for C2P10, while in Pitrufquén the highest value was also 3932.08 kg ha^−1^ for C3P5. In Temuco, C1P19 reached 8526.63 kg ha^−1^, followed by C2P18 (4765.51 kg ha^−1^) and C3P19 (3739.75 kg ha^−1^). In Valdivia, the highest-yielding accessions were C3P9 (5000.58 kg ha^−1^) and C3P13 (4209.89 kg ha^−1^). Fruit number per plant ranged from 0 to approximately 7200 fruits (Figure 2C), with the highest frequency concentrated between 0 and 600 fruits. At the accession level (Figure 2D), the maximum value in Nueva Imperial was 7017 fruits for C4P8 (not classified as an outlier). In Río Bueno, C4P6 was the only outlier with 4733 fruits. In Temuco, C4P20 reached 6709 fruits, while in Valdivia, C2P15 (4339 fruits) and C1P14 (3853 fruits) were identified as outliers. Fruit diameter distributions (Figure 2E) indicated that most fruits ranged between 3.9 and 8.1 mm, with a dominant peak around 6.9–7.2 mm. The largest fruit diameter was recorded for C4P20 (8.0 mm; Temuco) (Figure 2F). Total soluble solids (°Brix) displayed wide dispersion (Figure 2G), with most values concentrated around 14–16 °Brix, although higher values (10–30 °Brix) also occurred. At the accession level (Figure 2H), the highest °Brix values were recorded in Río Bueno (C2P3, 27 °Brix) and Temuco, where C1P22 reached 29 °Brix, followed by C4P19 (28 °Brix) and C3P20 (27 °Brix).

#### 2.2.2. Evaluation of Productive Traits Under Rainfed Conditions

**Figure 3:**
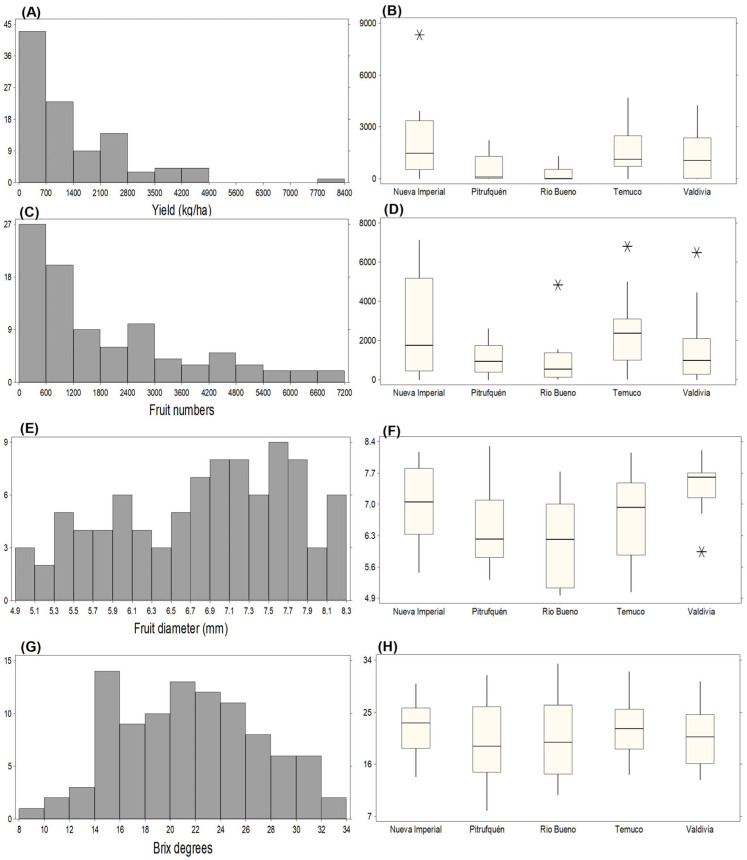
Distribution and variation of yield and fruit traits across different locations on 2022/2023 season. (**A**) Frequency distribution of yield. (**B**) Yield across locations. (**C**) Frequency distribution of fruit numbers. (**D**) Fruit numbers across locations. (**E**) Frequency distribution of fruit diameter. (**F**) Fruit diameter across locations. (**G**) Frequency distribution of Brix degrees. (**H**) Brix degrees across locations. Extreme values are indicated as “*” (possible outliers), as defined in the statistical analysis section.

Under rainfed conditions during the 2022/2023 season, yield per hectare again exhibited wide variability (Figure 3A,B), with most accessions producing <700 kg ha^−1^. Accession C4P7 (Nueva Imperial) strongly deviated from the population distribution, reaching 8334.30 kg ha^−1^ and representing the highest-yielding genotype (Figure 3B). Fruit number showed a comparatively more uniform distribution (Figure 3C), with the highest frequency again concentrated between 0 and 600 fruits plant^−1^. However, the boxplot identified multiple high outliers (Figure 3D), including C4P8 (7120 fruits; Nueva Imperial), C4P6 (4836 fruits; Río Bueno), C4P20 (6812 fruits; Temuco), and C3P14 (6490 fruits; Valdivia). Fruit diameter under rainfed conditions ranged from 4.9 to 8.3 mm (Figure 3E), with peaks around 7.1–7.5 mm and 7.9 mm.

The largest fruits were recorded in C3P1 (8.16 mm; Nueva Imperial), C2P14 (8.29 mm; Pitrufquén), C2P22 (8.15 mm; Temuco), and C1P12 (8.20 mm; Valdivia) (Figure 3F). Soluble solids also showed a broad distribution, with peaks around 14 and 28 °Brix (Figure 3G). The highest values were observed in C3P5 (31.33 °Brix; Pitrufquén), C3P16 (33.37 °Brix; Río Bueno), and C4P24 (32 °Brix; Temuco) (Figure 3H). When compared descriptively with the irrigated season, total fresh fruit production increased by 22.21% under rainfed conditions, total fruit number increased from 144,542 to 179,096 (+23.90%), average fruit diameter increased by 10.57%, and soluble solids increased by 34.87%. Because irrigation regime coincided with season and plant age and was not experimentally separated, these differences should be interpreted as seasonal contrasts rather than isolated irrigation effects.

### 2.3. Evaluation of Nutritional Value

To evaluate the nutritional potential of Michay fruit, a biochemical characterization was conducted including proximate composition, antioxidant activity (ORAC), and quantification of total polyphenols and anthocyanins. These variables were selected to capture both macronutritional composition and functional antioxidant attributes across contrasting accession profiles. Proximate composition provides information on proteins, lipids, carbohydrates, dietary fiber, and energy content, whereas ORAC reflects the capacity of extracts to scavenge peroxyl radicals. Total polyphenols and anthocyanins were included due to their well-established contribution to antioxidant activity.

#### 2.3.1. Proximate Composition and Antioxidant Activity of Michay Fruit

The proximate composition of Michay fruits, expressed on a dry weight basis, showed marked variability among selected accessions representing contrasting productive and antioxidant profiles (Table 3). For descriptive purposes, three accessions were selected based on productivity (high: C4P7; medium: C1P20; low: C1P3) and three based on ORAC activity (high: C3P11; medium: C3P17; low: C4P7). Notably, accession C4P7 represented a high-yielding but low-ORAC profile, and was therefore included in both groupings to highlight contrasting productive and functional performance. Protein content ranged from 13.78% (low-yielding accession) to 17.12% (medium-yielding accession). Within the antioxidant-based grouping, protein content was highest in the low-ORAC accession (16.17%), followed by the high-ORAC (13.83%) and medium-ORAC (10.36%) accessions. Fat content showed a similar pattern: the medium-yielding accession exhibited the highest value (11.91%) and the low-yielding accession the lowest (6.48%). In the antioxidant grouping, fat content was higher in the low-ORAC accession (8.43%) than in the medium-ORAC (5.87%) and high-ORAC (4.84%) accessions. Dietary fiber varied substantially among accessions. The high-ORAC accession exhibited the highest fiber content (55.44%), whereas medium- and low-ORAC accessions showed lower values (35.19–43.12%). Among productivity-based accessions, fiber content was highest in the high-yielding accession (43.12%), with similar values in the medium- and low-yielding accessions (39.04% and 40.43%, respectively). Ash content remained comparatively stable across accessions (3.15–3.51%). Carbohydrate content differed markedly among accessions, being highest in the medium-ORAC accession (45.54%) and lowest in the high-ORAC accession (22.49%). Similarly, carbohydrate content was highest in the low-yielding accession (36.16%), whereas high- and medium-yielding accessions showed comparable values (~28.74%). Energy content followed these trends, with the highest values recorded in the medium-yielding (290.6 kcal 100 g^−1^) and medium-ORAC (276.3 kcal 100 g^−1^) accessions, and the lowest value in the high-ORAC accession (188.8 kcal 100 g^−1^). Overall, these results indicate substantial quantitative differences in macronutrient composition among accessions selected for contrasting productive and functional profiles, supporting subsequent multivariate integration and interpretation.

#### 2.3.2. Concentration of Total Polyphenols, Anthocyanins, and ORAC Activity in Michay Fruit Under Irrigated Conditions

**Figure 4:**
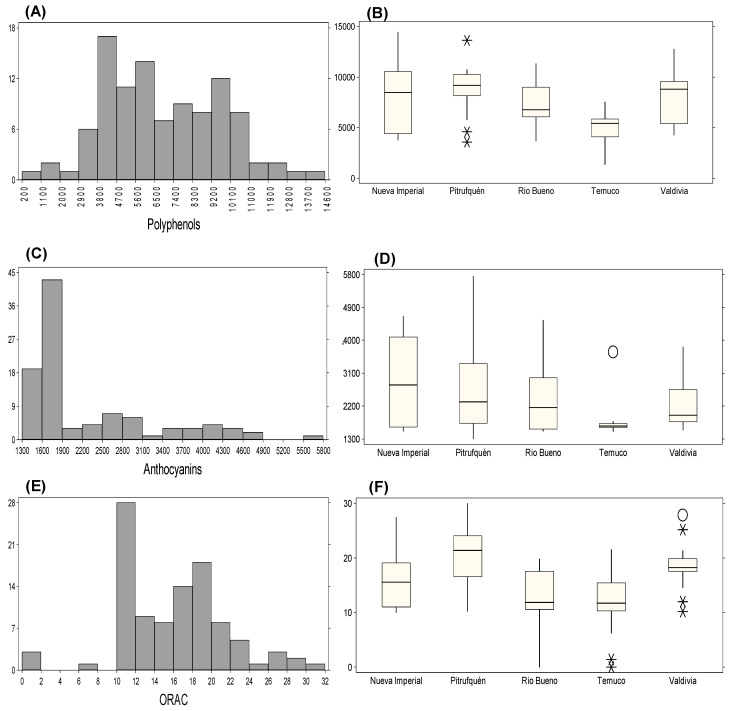
Distribution and variation of bioactive compounds and antioxidant capacity across different locations in 2021/2022 season. (**A**) Frequency distribution of total polyphenols. (**B**) Total polyphenols across locations. (**C**) Frequency distribution of total anthocyanins. (**D**) Total anthocyanins across locations. (**E**) Frequency distribution of ORAC values. (**F**) ORAC values across locations. Extreme values are indicated as “*” (possible outliers) and “O” (probable outliers), as defined in the statistical analysis section.

The chemical composition of Michay fruits showed clear differences between the two evaluated seasons (Table 4). Total polyphenols increased from 7017.50 mg GAE 100 g^−1^ in 2021/2022 season to 7607.80 mg GAE 100 g^−1^ in 2022/2023 season, accompanied by a higher standard deviation and a slight increase in relative variability (CV: 38.78% to 42.55%). Total anthocyanins also increased from 2266.40 to 2790.60 mg cyanidin-3-glucoside 100 g^−1^, while variability decreased markedly (CV: 43.04% to 25.66%), indicating a more homogeneous anthocyanin distribution across accessions in 2022/2023. ORAC antioxidant activity increased from 15.50 to 23.28 mmol Trolox 100 g^−1^ fw, with a decrease in CV (36.28% to 31.77%), reflecting higher antioxidant capacity with moderately reduced relative variability. Histogram and boxplot analyses supported these seasonal shifts and identified accessions with extreme values (Figure 4). During 2021/2022, polyphenol concentrations were most frequently distributed between 3800 and 4700 mg GAE 100 g^−1^ (Figure 4A), with the highest values in C4P8 (Nueva Imperial: 14,438.50 mg GAE 100 g^−1^), C1P13 (Pitrufquén: 13,626.90 mg GAE 100 g^−1^), and C3P13 (Valdivia: 12,768.56 mg GAE 100 g^−1^) (Figure 4B). Anthocyanins were most frequently distributed around 1600–1900 mg cyanidin-3-glucoside 100 g^−1^ (Figure 4C), while exceptionally high values were observed in C4P12 (Pitrufquén: 5747.76 mg 100 g^−1^), C4P13 (Nueva Imperial: 4649.09 mg 100 g^−1^), C1P7 (Río Bueno: 4546.60 mg 100 g^−1^), C4P8 (Temuco: 3674.89 mg 100 g^−1^), and C3P13 (Valdivia: 3813.30 mg 100 g^−1^) (Figure 4D). ORAC values were most frequently observed between 10 and 12 mmol TE 100 g^−1^ (approximately 28 accessions) (Figure 4E), with the highest values in C1P15 (Pitrufquén: 29.94 mmol TE 100 g^−1^), C1P17 (Nueva Imperial: 27.41 mmol TE 100 g^−1^), C4P18 (Temuco: 21.50 mmol TE 100 g^−1^), C3P11 (Río Bueno: 19.80 mmol TE 100 g^−1^), and the outliers C1P10 (27.84 mmol TE 100 g^−1^) and C2P5 (25.13 mmol TE 100 g^−1^) from Valdivia (Figure 4F). Overall, these results document substantial inter-accession variability and seasonal shifts in polyphenol and anthocyanin accumulation, as well as antioxidant capacity, under irrigated conditions.

#### 2.3.3. Concentration of Total Polyphenols, Anthocyanins, and ORAC Activity in Michay Fruit Under Rainfed Conditions

**Figure 5:**
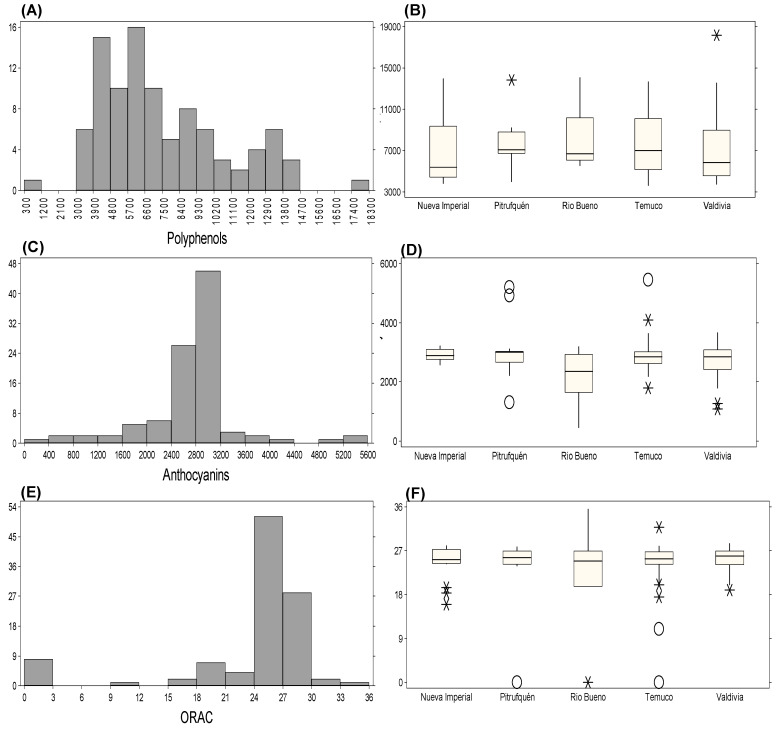
Distribution and variation of bioactive compounds and antioxidant capacity across different locations in 2022/2023 season. (**A**) Frequency distribution of total polyphenols. (**B**) Total polyphenols across locations. (**C**) Frequency distribution of total anthocyanins. (**D**) Total anthocyanins across locations. (**E**) Frequency distribution of ORAC values. (**F**) ORAC values across locations. Extreme values are indicated as “*” (possible outliers) and “O” (probable outliers), as defined in the statistical analysis section.

Under rainfed conditions during the 2022/2023 season, histograms and boxplots revealed distinct biochemical performance across the 96 accessions (Figure 5). Polyphenols showed a right-skewed distribution, with most accessions ranging between 5700 and 6600 mg GAE 100 g^−1^ and a marked decline in frequency beyond 10,200 mg GAE 100 g^−1^ (Figure 5A). Several accessions reached substantially higher values, including C2P14 (Pitrufquén: 13,855.00 mg GAE 100 g^−1^), C1P8 (Río Bueno: 14,072.98 mg GAE 100 g^−1^), and C1P12 (Valdivia), which recorded the maximum concentration (18,168.73 mg GAE 100 g^−1^) (Figure 5B). Anthocyanin concentrations were most frequently between 2800 and 3200 mg cyanidin-3-glucoside 100 g^−1^ (Figure 5C), with exceptionally high values in C3P6 (5204.24 mg 100 g^−1^) and C1P2 (4917.51 mg 100 g^−1^) from Pitrufquén, and in C1P25 (Temuco: 5454.47 mg 100 g^−1^) and C2P23 (Temuco: 4096.52 mg 100 g^−1^) (Figure 5D). ORAC activity shifted toward higher values relative to the irrigated season, with the most frequent interval between 24 and 27 mmol TE 100 g^−1^ (approximately 52 accessions) (Figure 5E). The highest ORAC was recorded in C3P11 (Río Bueno: 35.49 mmol TE 100 g^−1^), followed by C1P25 (Temuco: 31.78 mmol TE 100 g^−1^), which was classified as an outlier (Figure 5F). When biochemical responses were compared descriptively across seasons, total polyphenols increased from 7017.50 to 7607.01 mg GAE 100 g^−1^, total anthocyanins increased by 23.12%, and ORAC activity increased by 59.49% under rainfed conditions relative to the irrigated 2021/2022 season. Because irrigation regime coincided with plant age and seasonal conditions and was not independently replicated, these differences should be interpreted as seasonal contrasts rather than isolated water-deficit effects. Overall, these results highlight pronounced inter-accession variability and season-dependent shifts in antioxidant-related compounds in *B. darwinii* fruits under rainfed conditions.

### 2.4. Multivariate Integration of Morphological, Productive, and Biochemical Traits

**Figure 6:**
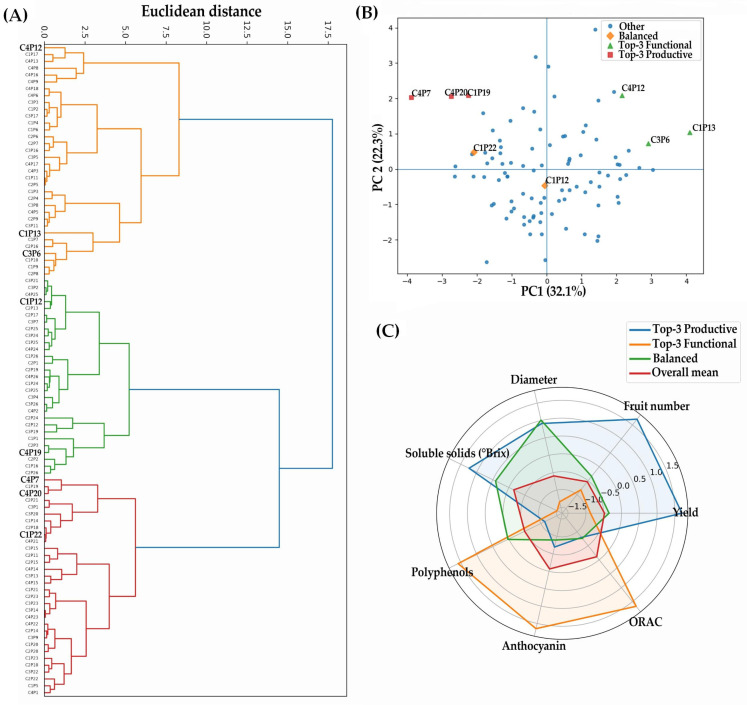
Multivariate identification of elite *B. darwinii* accessions based on integrated productive and functional traits. (**A**) Hierarchical clustering of the 96 accessions based on Euclidean distances calculated from standardized principal component analysis (PCA) scores. (**B**) Principal component analysis biplot showing the distribution of accessions. Accessions identified as top-3 productive, top-3 functional, and balanced are highlighted, while remaining accessions are shown in blue. (**C**) Radar plot comparing standardized multi-trait profiles of accession groups, including top 3 productive, top 3 functional, balanced accessions, and the overall population mean.

**Figure 7:**
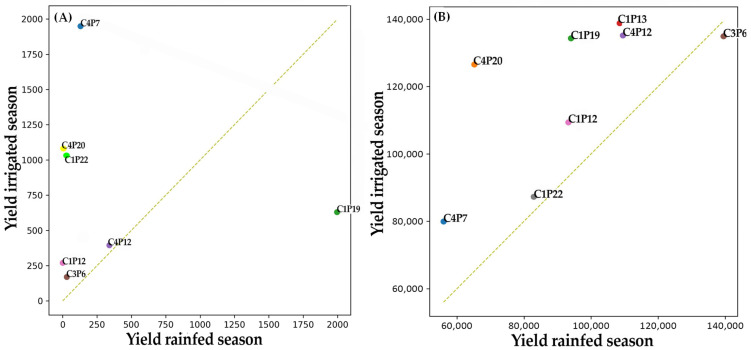
Seasonal stability of elite *Berberis darwinii* accessions. (**A**) Yield per plant under irrigated season versus rainfed season. (**B**) ORAC antioxidant capacity under irrigated season versus rainfed season.

To integrate morphological, productive, and biochemical variation across *B. darwinii* accessions, hierarchical clustering was performed using Euclidean distances computed from standardized principal component analysis (PCA) scores. The resulting dendrogram (Figure 6A) revealed a clear multivariate structure, separating the 96 accessions into three major groups broadly corresponding to: (i) a cluster dominated by productive traits, (ii) a cluster dominated by functional/biochemical traits, and (iii) an intermediate group characterized by balanced multi-trait performance. Notably, clustering was not associated with geographic origin, indicating that differentiation reflected intrinsic phenotypic and biochemical variation maintained under common-garden conditions. To identify the trait combinations underlying these groups, PCA was conducted using standardized (z-score) morphological, productive, and biochemical variables. The first two components explained 54.4% of total variance (PC1 = 32.1%, PC2 = 22.3%; Figure 6B). Trait loadings and correlation structure (Figure 6B; Table 5) indicated that PC1 was mainly associated with productive performance (yield per plant, fruit number, fruit diameter, and soluble solids, °Brix), whereas PC2 was primarily driven by biochemical and functional traits (total polyphenols, anthocyanins, ORAC antioxidant capacity, and dietary fiber). Morphological traits, including shoot number and foliar area, showed intermediate contributions, linking vegetative vigor with both productive and functional dimensions. The PCA score plot showed wide dispersion across the multivariate space and again revealed no clear grouping by geographic origin, confirming persistent differentiation under common-garden conditions. Several accessions occupied extreme positions and defined contrasting multi-trait syndromes. Accessions at the negative extreme of PC1 were characterized by high yield and fruit number, identifying a productive elite group including C4P7, C4P20, and C1P19. In contrast, accessions at the positive extreme of PC2 exhibited high polyphenol and anthocyanin concentrations together with elevated ORAC activity, defining a functional elite group represented by C4P12, C3P6, and C1P13. A subset of accessions, notably C1P12 and C1P22, occupied intermediate positions close to the PCA origin, reflecting a balanced profile with moderate-to-high values across both productive and functional traits. Concordance between the PCA ordination and hierarchical clustering supports the robustness of these multivariate patterns. Multi-trait radar profiles (Figure 6C), integrating standardized values for yield, fruit number, fruit diameter, soluble solids, polyphenols, anthocyanins, and ORAC activity, further summarized these syndromes. Radar plots illustrated clear contrasts between productive and functional elite groups, while highlighting balanced accessions as non-extreme but consistently multi-dimensional performers relative to the population mean. Finally, trait stability across seasons provided an additional dimension for evaluating elite accessions. Yield stability analysis (Figure 7A) showed that productive elite accessions maintained high yields across seasons, whereas balanced accessions displayed moderate but stable productivity. Similarly, ORAC stability analysis (Figure 7B) indicated that functional elite accessions consistently expressed high antioxidant capacity, while balanced accessions maintained intermediate yet stable ORAC values. Because seasonal contrasts also encompassed plant developmental stage and were not experimentally separated from the irrigation regime, stability patterns should be interpreted as temporal consistency across seasons rather than isolated water-regime effects. Overall, these seasonal comparisons support that elite status was not driven solely by extreme performance in a single season, but was reinforced by temporal consistency under contrasting seasonal conditions.

## 3. Discussion

The present study provides the first comprehensive experimental evaluation of phenotypic, productive, and biochemical diversity in *B. darwinii* cultivated under common-garden conditions and contrasting seasonal water availability. Crucially, our goal is not a descriptive germplasm screening per se, but to interpret intra-specific variation through a trait-integration lens, showing how productivity and fruit-quality traits relate to phenolic/anthocyanin accumulation and antioxidant capacity under common-garden cultivation. This supports a trait-based selection framework and provides evidence for coordinated phenotypic and metabolic syndromes in a perennial shrub. The coordinated variation observed among productive, morphological, and biochemical traits reflects structured phenotypic diversity rather than random accession-level variation, supporting the relevance of trait-based selection frameworks. The magnitude of morphological variation detected across accessions confirms that Michay exhibits pronounced phenotypic plasticity, consistent with reports for wild populations and related Berberis species adapted to heterogeneous environments [38,39,40,41]. Traits such as shoot number, plant height, and leaf area displayed high coefficients of variation, indicating substantial genetic diversity that can be harnessed for selection and breeding. In particular, shoot number and leaf area, which are closely linked to canopy development and photosynthetic capacity, emerged as key contributors to productive potential, as also suggested for woody shrub crops adapted to stress-prone environments [39]. Productive performance varied markedly among accessions and between seasons, reinforcing the importance of genotype-dependent responses under low-input conditions. Although irrigation, plant age, and seasonality were confounded, the overall improvement in yield, fruit number, fruit diameter, and soluble solids observed in the between-season contrast indicates that a substantial proportion of Michay accessions are capable of maintaining or even enhancing productivity under low-input cultivation scenarios. This contrasts sharply with other Chilean native berries, such as *U. molinae*, *B. microphylla*, and *A. chilensis*, which show marked yield reductions, increased mortality, or strong dependence on irrigation under similar conditions [34,35,36,42]. The ability of Michay to sustain yields above 8 t ha^−1^ in the 2022/2023 seasonal contrast, characterized by rainfed conditions, places it within the range of commercial berry crops, while requiring substantially lower applied irrigation inputs than conventional systems such as blueberry production [30]. Fruit quality traits followed a similar pattern, with soluble solids showing strong enhancement in the between-season comparison. Increased °Brix values are commonly associated with concentration effects under moderate water stress, but often at the expense of fruit size or yield [36]. In Michay, however, higher °Brix coincided with stable or increased fruit diameter and fruit number, indicating that, in the seasonal contrast evaluated, higher soluble solids did not coincide with reductions in fruit diameter or yield, suggesting the absence of a classical yield–quality trade-off under the conditions studied. This coordinated response suggests efficient carbon allocation and osmotic adjustment mechanisms that allow sugar accumulation without compromising reproductive output, a trait of high agronomic relevance for low-input berry systems. Biochemical analyses further revealed that antioxidant-related traits were strongly influenced by seasonal growing conditions. In berry crops, moderate water deficit is often associated with increased accumulation of phenolic compounds, including anthocyanins, due to activation of secondary metabolism and enhanced carbon allocation toward protective pathways. However, such biochemical enhancement frequently occurs at the expense of fruit size, yield, or overall plant vigor, reflecting classical stress-induced trade-offs reported in species such as *Vaccinium* spp., *Fragaria* spp., and other perennial fruit crops. In contrast, the present results suggest that *B. darwinii* may exhibit a distinct response pattern in the seasonal water-availability contrast evaluated here, where antioxidant accumulation did not coincide with marked reductions in productivity. Total polyphenols, anthocyanins, and ORAC activity increased in the seasonal water-availability contrast, although this cannot be attributed solely to irrigation due to the coinciding effects of season and plant age, with ORAC showing the largest relative enhancement. Similar increases in antioxidant compounds have been reported for native berries and other perennial crops exposed to abiotic stress [5,6,36], reflecting the activation of secondary metabolism as part of acclimation strategies. Importantly, these biochemical gains did not occur at the expense of productivity in the seasonal contrast evaluated, highlighting Michay’s capacity to combine low-input performance with functional quality. This coordinated maintenance of yield and antioxidant potential suggests a potentially advantageous physiological resilience and supports the relevance of this species for climate-resilient breeding strategies targeting low-input production systems. Notably, several accessions reached ORAC values exceeding the upper ranges reported for maqui, murtilla, and calafate in the literature [5,6]. As shown in Table 4, total polyphenols reached up to 18,168.73 mg GAE 100 g^−1^ dw in accession C1P12 (Valdivia), anthocyanins reached 5747.76 mg C3G 100 g^−1^ dw in accession C4P12 (Pitrufquén), and ORAC values peaked at 35.49 mmol TE 100 g^−1^ fw in accession C3P11 (Río Bueno). Although coefficients of variation were moderate to high (CV ranging from 25.66% to 42.55%), this variability reflects substantial intra-specific diversity across the 96 evaluated accessions rather than isolated extreme observations, supporting the robustness of the observed antioxidant potential. While direct comparisons among studies should be interpreted cautiously due to methodological and environmental differences, these results highlight the remarkable antioxidant capacity of *B. darwinii* and reinforce its relevance for functional food and nutraceutical applications. Phenolic-rich extracts from native berries have been associated with anti-inflammatory, anti-diabetic, and cardioprotective biological effects in previous studies. While the present work focuses on biochemical characterization rather than direct bioactivity assays, the identification of elite accessions with elevated phenolic and antioxidant profiles provides a germplasm foundation for downstream validation of biological effects. The integration of morphological, productive, and biochemical variables through PCA provided a robust framework for identifying elite accessions with contrasting and complementary trait syndromes. PCA clearly separated accessions oriented toward high productivity from those characterized by superior antioxidant profiles, while also revealing a subset of balanced genotypes that combined moderate-to-high yield with elevated functional attributes. This multivariate structure supports the concept of multiple breeding targets rather than a single “ideal” genotype, allowing selection strategies to be tailored to specific end uses, such as fresh markets, processing, or functional ingredient development. Importantly, the identification of top-performing accessions within each functional group underscores the feasibility of selective domestication in Michay. High-yielding accessions demonstrated productivity levels compatible with commercial cultivation, while high-antioxidant accessions exhibited biochemical profiles suitable for nutraceutical and functional food applications. Balanced accessions, which did not maximize a single trait but showed stable intermediate performance across productivity and antioxidant dimensions, represent particularly valuable material for low-input and climate-adaptive systems, where stability across seasons is often more desirable than maximum performance under optimal conditions. Taken together, these results provide experimental evidence supporting Michay’s adaptive performance and confirm that its phenotypic and biochemical diversity can be strategically exploited through selection rather than genetic modification or intensive management. The combination of high antioxidant potential and strong performance in the seasonal contrast characterized by rainfed conditions, and wide intra-specific variability supports the domestication of *B. darwinii* as a novel berry crop aligned with the goals of sustainable agriculture, climate adaptation, and functional food development.

## 4. Materials and Methods

### 4.1. Establishment of Plant Material and Experimental Design

A common-garden experiment was established at the Huichahue Experimental Field of the Agriaquaculture Nutritional Genomics Center (CGNA), in the Padre Las Casas commune (E 721481; N 5698107). Wild Michay plants were collected in their native habitats in June 2019 across five zones in southern Chile: Temuco (34 plants), Pitrufquén (13), Nueva Imperial (17) in the La Araucanía Region, and Río Bueno (14) and Valdivia (18) in the Los Ríos Region. Each plant was established as an individual accession representing a unique wild genotype, and all accessions were maintained under identical field conditions. The orchard was planted with north–south orientation, establishing four ridges (40 cm high × 50 cm wide) with spacing of 2.6 m between rows and 0.9 m between plants, allowing the establishment of 24 plants per ridge for a total of 96 accessions (planting density: 4274 plants ha^−1^). Plants were grouped in sets of three according to their geographical origin and randomly assigned to positions across ridges. Each individual plant constituted the experimental unit. The orchard was enclosed with open mesh for protection, and a black weed-control mesh was installed on each ridge. The soil at the site corresponds to the Temuco series (Andisols), derived from ancient volcanic ash deposited on remnant plains, typically found in the central valley at 100–300 m a.s.l. with a dark brown loamy-clay surface texture. Soil chemical properties prior to establishment were: N = 16 mg kg^−1^, P = 7 mg kg^−1^, K = 137 mg kg^−1^, Mn = 0.64 mg kg^−1^, Zn = 0.21 mg kg^−1^, Cu = 0.52 mg kg^−1^, Fe = 28 mg kg^−1^, B = 0.3 mg kg^−1^, pH = 6.1, and organic matter = 17%. Before planting, the soil was plowed to 40 cm depth, harrowed, and mounded to a height of 40 cm.

### 4.2. Agronomic Management

The experiment was conducted over two consecutive growing seasons. An irrigation system was installed on each of the four ridges using 26 m irrigation tapes with built-in self-compensating drippers (Netafim), operating at a flow rate of 4 L h^−1^ and spaced 0.6 m apart. Two irrigation lines were placed per row, providing two drippers per plant to ensure uniform water distribution. Plants were irrigated for 40 min every other day, totaling four irrigation events per week. Once the accessions reached morphological and productive stability, as determined from repeated evaluations initiated in 2019, two contrasting water availability regimes were evaluated across two consecutive seasons: (1) full drip irrigation for all accessions from September to February in the 2021/2022 season, and (2) rainfed conditions for all plants in the 2022/2023 season. Because irrigation regime, season, and plant age coincided, this comparison represents contrasting seasonal water availability rather than a replicated factorial irrigation treatment. No fertilizers, fungicides, or pesticides were applied during either season. Meteorological data, including temperature (°C; average, maximum, and minimum), precipitation (mm), evapotranspiration (mm), chill hours (h), degree days (base 10; –day), and frost events, were recorded from the Huichahue weather station (Effitrade, Chile) operated by CGNA (Padre Las Casas, −38.05464, −72.76329) (Table 6).

### 4.3. Evaluation of Morphological Traits

All 96 Michay accessions were evaluated for growth habit (erect or semi-erect), plant height (cm), shoot number, foliar area (cm^2^), and number of spines per leaf. Growth habit was visually determined based on branch bending angle: plants whose branches bent more than 90° were classified as semi-erect, whereas those maintaining a vertical branch position were classified as erect. Plant height was measured using a measuring tape. For foliar area determination, 50 leaves per plant were collected from the four cardinal directions and representative canopy heights. Leaf area was measured immediately after collection to avoid tissue deformation; samples were not frozen or stored prior to scanning. Foliar area was quantified using ImageJ 1.42 (Wayne Rasband, National Institutes of Health, Bethesda, MD, USA). Shoot number and number of spines (50 leaves per plant) were quantified by direct counting [43].

### 4.4. Evaluation of Productive Traits

All fruits produced by each individual plant during the entire growing season were harvested and pooled. Fruits were weighed and counted to determine total yield and fruit number. Subsequently, 50 fruits per accession were randomly selected and measured for diameter (mm) using a caliper (Maxcel, Valent, Santiago, Chile). Additionally, juice from 50 fruits per accession was analyzed using a refractometer (Hanna Instruments HI96801, Woonsocket, RI, USA) to determine total soluble solids (°Brix) [44].

### 4.5. Evaluation of Antioxidant Content and Activity

Total polyphenols, anthocyanins, and antioxidant capacity (ORAC) were quantified to characterize the functional properties of Michay fruits. All biochemical analyses were performed in triplicate per accession.

#### 4.5.1. Extraction Procedure

One kilogram of fresh fruit was lyophilized for 3 days and subsequently pulverized to 0.2 mm (Frittsch, Pulverisette 14, Oberstain, Germany). For the extraction procedure, 20 mg of powdered material was weighed and dissolved in 1 mL of 80% (*w*/*w*) ethanol. Samples were vigorously shaken at maximum power for 3 h using a multivortex mixer (Heidolph, Multi Reax, Schwabach, Germany) and then centrifuged for 5 min at 13,000 rpm (Eppendorf AG, 5810 R, Hamburg, Germany). After centrifugation, the supernatant was transferred to a clean container and stored at 4 °C for subsequent analyses [45].

#### 4.5.2. Determination of Total Polyphenols

Total polyphenols were quantified using the Folin–Ciocalteu method [46]. Extracts were diluted 1:9 with 80% (*w*/*w*) ethanol and mixed with Folin–Ciocalteu reagent, followed by vortexing and incubation at room temperature for 30 min in darkness. Samples were then centrifuged for 5 min at 13,000 rpm and transferred to a microplate. Absorbance was measured at 750 nm using a Multi-Detection Microplate Reader (Biotek, Synergy HT Instruments Inc., Winooski, VT, USA). Gallic acid was used as the external calibration standard. Standard solutions were prepared in the range of 0–500 mg L^−1^ to construct a linear calibration curve (R^2^ = 0.995; slope = 0.00089). All sample absorbance values fell within the linear range of the standard curve. Total polyphenols were expressed as mg gallic acid equivalents per 100 g dry weight (mg GAE 100 g^−1^ dw) [47]. All analyses were performed in triplicate.

#### 4.5.3. Determination of Total Anthocyanin

Total anthocyanins were quantified using the pH differential method [47]. For sample preparation, 0.5 g of pulp was weighed and mixed with 25 mL of 0.1% (*w*/*v*) HCl. Samples were vigorously shaken on a multivortex for 10 min and centrifuged at 4000 rpm for 10 min. This extraction procedure was repeated four times, and the resulting extracts were stored at 4 °C. For dilution and analysis, the first extract was diluted 1:4, and 80 μL of the diluted solution was mixed with 1100 μL of pH 1.0 KCl buffer. Subsequent extracts were diluted 1:2 by combining 590 μL of sample with 590 μL of pH 1.0 KCl buffer. Each tube was thoroughly mixed, and 300 μL of each preparation was transferred to a microplate in triplicate for absorbance readings at 515 nm and 700 nm. The entire analytical procedure was then repeated using pH 4.5 sodium acetate buffer (NaCH_3_COO).

Anthocyanin concentration was calculated using the pH differential formula:

A = (A515 − A700) pH 1.0 − (A515 − A700) pH 4.5,

and expressed as cyanidin-3-O-glucoside equivalents using a molecular weight of 449.2 g mol^−1^ and a molar extinction coefficient (ε) of 26,900 L mol^−1^ cm^−1^. Results were expressed as mg cyanidin-3-glucoside equivalents per 100 g dry weight (mg C3G 100 g^−1^ dw).

#### 4.5.4. Determination of ORAC Activity

Antioxidant capacity was measured using the oxygen radical absorbance capacity (ORAC) assay following the protocol described by Ou et al. [48]. Five µL of extract was diluted in 995 µL of ORAC working solution (Sigma-Aldrich, St. Louis, MO, USA). Then, 25 µL of fluorescein were added to each well of the microplate and incubated for 30 min at 45 °C. Subsequently, 25 µL of AAPH was added to initiate the reaction, and fluorescence decay was monitored for 90 min at 1 min intervals (excitation 485/20 nm; emission 520 nm). Trolox was used as the external calibration standard. A stock solution (4834.39 µmol L^−1^) was prepared, and calibration points were generated by adding 5, 10, 20, and 40 µL of the standard solution to the reaction mixture. The calibration curve showed strong linearity (R^2^ = 0.995; slope = 0.1566; intercept = 0.0286). ORAC values were calculated from the net area under the fluorescence decay curve and expressed as mmol Trolox equivalents per 100 g fresh weight (mmol TE 100 g^−1^ fw). All analyses were performed in triplicate.

### 4.6. Proximate Composition

Protein content was quantified using the Dumas method (Dumatherm^®^ N PRO analyzer, Königswinter, Germany) with a conversion factor of 6.25. Oil content was determined according to the AOAC Official Method 920.39. Total dietary fiber was analyzed using the enzymatic–gravimetric method [45]. Moisture content was determined via the oven-drying gravimetric method at 105 °C (NCh 841 Of. 78). Carbohydrate content (nitrogen-free extract) was calculated by difference. All proximate composition results were expressed on a dry weight basis (dw).

### 4.7. Statistical Analyses

Statistical analyses were performed using Statistix 10 (Tallahassee, FL, USA). Analysis of variance (ANOVA) followed by Tukey’s test was applied to compare yield, ORAC, and all proximate composition variables. For each morphological, productive, and nutritional trait, tables were generated that included the coefficient of variation. To identify outlier accessions, boxplots were constructed using a box bisected by the median, with two whiskers. Extreme values were represented as “*” for possible outliers and “O” for probable outliers. Possible outliers were defined as observations located more than 1.5 times the interquartile range from either boundary, whereas probable outliers were values located more than three times the interquartile range away. Data normality and homoscedasticity were verified prior to statistical inference. A significance level of *p* ≤ 0.05 was used for all analyses. In addition to univariate analyses, Pearson correlation analysis was performed among key morphological (leaf area, shoot number), productive (fruit number, yield, fruit diameter, °Brix), and biochemical (total polyphenols, anthocyanins, ORAC, fiber, protein) variables to identify trait associations and potential trade-offs. Multivariate structure was explored using Principal Component Analysis (PCA) based on standardized (z-score) morphological, productive, and biochemical variables to integrate trait syndromes rather than test causal relationships, and to identify elite accessions showing multi-trait superiority. The experimental unit for all analyses was the individual plant. Because irrigation regime coincided with season and plant age and was not independently replicated, comparisons between seasons should be interpreted as seasonal contrasts rather than isolated irrigation effects.

## 5. Conclusions

This study identified a set of elite *Berberis darwinii* (Michay) accessions with clear potential for use in breeding programs targeting both fruit productivity and functional quality. In the 2021/2022 irrigated season, accession C1P19 emerged as the most productive genotype, combining high yield with favorable fruit traits, and thus represents a strong candidate for productivity-oriented selection. Accession C4P8 stood out for its consistently high fruit number and elevated total polyphenol content, highlighting its value for applications focused on bioactive compound production. In the 2022/2023 seasonal contrast, characterized by rainfed conditions, C3P11 exhibited exceptional ORAC activity, representing a high-antioxidant profile under contrasting seasonal water availability. Similarly, C1P25 showed outstanding anthocyanin accumulation together with stable productive performance in the between-season comparison. Beyond individual elite accessions, the results reveal that Michay as a species expresses substantial phenotypic plasticity, productive capacity, and biochemical diversity under common-garden cultivation. In the seasonal contrast evaluated here, productive and antioxidant-related traits were maintained or enhanced; however, these differences cannot be attributed solely to irrigation due to coinciding seasonal and developmental effects. This combination of productivity and functional quality under low-input management highlights the species’ potential for sustainable agriculture and value-added functional food applications. Overall, the integration of morphological, productive, and biochemical traits enabled the identification of accessions suited to distinct breeding objectives, including high yield, enhanced antioxidant capacity, and balanced multi-trait performance. Future research should validate these elite accessions across multiple environments, disentangle genotype × environment interactions, and assess postharvest behavior, processing performance, and sensory attributes to support the commercial domestication and deployment of *B. darwinii* in sustainable berry production systems.

## Figures and Tables

**Table 1:** Coefficient of variation for each morphological trait in Michay plants.

Morphological Traits	Mean	Standard Deviation	Coefficient of Variation (%)	Minimum	Maximum
Shoots (mean/plant)	11.32	4.82	42.60	1.00	25.00
Height (cm)	116.36	35.1	30.19	42.00	207.00
Spines (mean/leaf)	6.96	1.61	23.11	3.80	12.70
Foliar area (cm^2^)	1.07	0.36	34.06	0.37	2.01

**Table 2:** Coefficient of variation for each productive trait in Michay plants.

Productive Traits					
Season 2021/2022	Mean	Standard Deviation	Coefficient of Variation (%)	Minimum	Maximum
Yield (kg/ha)	1021.40	1459.40	142.89	0.00	8526.60
Diameter (mm)	6.15	0.80	13.11	4.07	8.00
Soluble solids (°Brix)	16.31	6.23	38.22	4.23	29.00
Number of fruits	1389.80	1701.90	122.45	0.00	7017.00
Season 2022/2023					
Yield (kg/ha)	1285.10	1451.80	112.97	0.00	8334.30
Diameter (mm)	6.81	0.91	13.40	4.97	8.29
Soluble solids (°Brix)	21.55	5.58	25.88	8.00	33.37
Number of fruits	1907.20	1828.10	95.84	3.00	7120.00

**Table 3:** Chemical composition of Michay fruits. Data are reported on a dry matter basis. ± corresponds to the standard deviation. Each analysis was performed in triplicate. The ecotypes used for measurements of productivity, diameter, soluble solids, and ORAC activity were selected according to the ranking from the highest to the lowest value, considering a maximum value, a minimum value, and an average value. For productivity, the evaluated accessions were, from highest to lowest, C4P7, C1P20, C1P3. And for ORAC activity, they were as follows: C3P11, C3P17, and C4P7. Different letters for macronutrients and energy within each category (yield or ORAC) indicate significant differences according to ANOVA followed by Tukey’s test.

Parameter	Yield (kg)			ORAC Activity		
	High	Medium	Low	High	Medium	Low
Protein (%)	16.17 ± 0.41 ^a^	17.12 ± 0.65 ^a^	13.78 ± 0.44 ^b^	13.83 ± 0.09 ^b^	10.36 ± 0.03 ^c^	16.17 ± 0.41 ^a^
Fat (%)	8.43 ± 0.33 ^b^	11.91 ± 0.09 ^a^	6.48 ± 0.25 ^c^	4.84 ± 0.04 ^c^	5.87 ± 0.13 ^b^	8.43 ± 0.33 ^a^
Dietary fiber (%)	43.12 ± 0.00 ^a^	39.04 ± 0.00 ^c^	40.43 ± 0.00 ^b^	55.44 ± 0.00 ^a^	35.19 ± 0.00 ^c^	43.12 ± 0.00 ^b^
Ash (%)	3.51 ± 0.05 ^a^	3.19 ± 0.00 ^b^	3.15 ± 0.02 ^b^	3.40 ± 0.01 ^b^	3.04 ± 0.03 ^c^	3.51 ± 0.05 ^a^
Carbohydrate available (%)	28.76 ± 0.00 ^b^	28.74 ± 0.00 ^b^	36.16 ± 0.00 ^a^	22.49 ± 0.00 ^c^	45.54 ± 0.00 ^a^	28.76 ± 0.00 ^b^
Energy (kcal/100g)	255.6 ^b^	290.6 ^a^	258.1 ^b^	188.8 ^c^	276.3 ^a^	255.6 ^b^

**Table 4:** Coefficient of variation for each nutritional trait in Michay plants.

Chemical Traits					
2021/2022 Season	Mean	Standard Deviation	Coefficient of Variation	Minimum	Maximum
Total polyphenols (mg GAE/100 g)	7017.50	2721.70	38.78	1364.00	14,439.00
Total anthocyanins (mg cyanidin 3 glucoside/100 g)	2266.40	975.62	43.04	1306.70	5747.80
ORAC activity (mmol Trolox/100 g fw)	15.50	5.62	36.28	0.00	29.94
**2022/2023 season**					
Total polyphenols (mg GAE/100 g)	7607.80	3237.60	42.55	3644.80	18,169.00
Total anthocyanins (mg cyanidin 3 glucoside/100 g)	2790.60	716.18	25.66	442.36	5454.50
ORAC activity (mmol Trolox/100 g fw)	23.28	7.39	31.77	0.00	35.49

**Table 5:** Pearson correlation matrix among morphological, productive, and biochemical traits of *Berberis darwinii* accessions. Values represent Pearson correlation coefficients (r). Positive values indicate direct relationships and negative values indicate inverse relationships between traits.

	Shoot Numbers	Height	Foliar Area	Spines	Yield	Fruit Number	Diameter	Soluble Solids	Polyphenols	Anthocyanins	ORAC
**Shoot numbers**	1	0.13	0	0.25	0.06	0.03	0.03	−0.01	0.11	0.07	0.2
**Height**	0.13	1	0.35	0.02	0.45	0.27	0.32	0.36	−0.12	−0.04	−0.08
**Foliar area**	0	0.35	1	0.41	0.11	−0.14	0.05	0.02	−0.15	−0.24	−0.05
**Spines**	0.25	0.02	0.41	1	−0.27	−0.27	−0.22	−0.01	0.06	−0.04	0.08
**Yield**	0.06	0.45	0.11	−0.27	1	0.57	0.31	0.23	−0.02	−0.07	−0.15
**Fruit number**	0.03	0.27	−0.14	−0.27	0.57	1	−0.07	0.09	0.06	0.07	−0.21
**Diameter**	0.03	0.32	0.05	−0.22	0.31	−0.07	1	0.34	−0.06	−0.01	−0.02
**Soluble solids**	−0.01	0.36	0.02	−0.01	0.23	0.09	0.34	1	−0.31	−0.04	−0.26
**Polyphenols**	0.11	−0.12	−0.15	0.06	−0.02	0.06	−0.06	−0.31	1	0.55	0.43
**Anthocyanins**	0.07	−0.04	−0.24	−0.04	−0.07	0.07	−0.01	−0.04	0.55	1	0.35
**ORAC**	0.2	−0.08	−0.05	0.08	−0.15	−0.21	−0.02	−0.26	0.43	0.35	1

**Table 6:** Climatic data for *Berberis darwinii*. ^1^ Minimum–Maximum, ^2^ Evapotranspiration and ^3^ Expressed in base 10.

Season	Mean Temperature (°C)	Temperature Min–Max ^1^ (°C)	Precipitation (mm)	ET ^2^ (mm)	Chill Hours (h)	Degree Days ^3^ (–Day)	Frost Events
**2021**	11.62	6.10–19.22	715.60	952.60	1717	1126	33
**2022**	11.39	6.03–18.29	1031.70	867.04	1887	1041	38
**2023**	11.71	5.58–18.14	820.50	871.44	1838	906	43

## Data Availability

The data presented in this study are available on reasonable request from the corresponding author. The data are not publicly available due to their relevance for ongoing research and breeding programs.
